# Tumor mutation burden associated with miRNA-gene interaction outcome mediates the survival of patients with liver hepatocellular carcinoma

**DOI:** 10.17179/excli2020-1224

**Published:** 2020-06-22

**Authors:** Qing-Jiang Yu, Yi-Zhi Liang, Xiao-Ping Mei, Tai-Yong Fang

**Affiliations:** 1 Department of Hepatobiliary Surgery, First Affiliated Hospital of Jiaxing University, Jiaxing 314000, China; 2 Department of Gastroenterology, Second Affiliated Hospital, Fujian Medical University, Quanzhou 362000, China

**Keywords:** tumor mutation burden (TMB), miRNA-gene interaction, liver hepatocellular carcinoma (LIHC), prognosis

## Abstract

Tumor mutation burden (TMB) is associated with immunogenic responses and the survival of cancer patients. This study demonstrates how TMB levels impact the immune-related cells, genes, and miRNAs, and how miRNA/gene interactions respond to variations in the survival rate of patients with liver hepatocellular carcinoma (LIHC). LIHC patients were divided into two groups, either a low TMB (< median) or a high TMB (≥ median) group. We found that high TMB plays a positive role in immune-mediated infiltration, generating more CD4 T-cells and memory B cells. Among the 21 immune genes that altered significantly, only *C9orf24* and *CYP1A1* were expected to up-regulate in LIHC patients with high TMB. A total of 19 miRNAs, which regulate various functional pathways, were significantly altered in patients with LIHC. One of the miRNA/gene pair, hsa-miR-33a/*ALDH1A3* was significantly associated with the survival rate of LIHC patients. Our results suggest that LIHC patients with high TMB can be treated more effectively with immunotherapy.

## Introduction

Liver cancer (LC) is the sixth most commonly diagnosed cancer, ranking the fourth in mortality among all cancer deaths worldwide in 2018 (Bray et al., 2018[3]). The highest incidence of LC is found in geographically heterogeneous regions including East and South-East Asia, and North and West Africa (Torre et al., 2015[43]; Yang et al., 2017[45]). Liver hepatocellular carcinoma (LIHC), generally known as hepatocellular carcinoma (HCC), accounts for 75 % - 85 % of all primary liver cancers (Bray et al., 2018[3]). Although progress is being made treating LIHC, its poor prognosis (high mortality rate) is still concerning (Yang et al., 2017[45]). 

Tumor mutation burden (TMB) is characterized by the somatic coding mutational landscape, involves the neoantigens of anti-tumor immunity, and is used in developing personalized cancer immunotherapy (Gubin et al., 2015[13]; Schumacher et al., 2015[35]; Schumacher and Schreiber, 2015[36]). Over the last few decades, an increasing number of studies have investigated TMB as an effective biomarker in several types of cancers (Kandoth et al., 2013[17]; Chalmers et al., 2017[5]; Vandeven et al., 2018[44]). Previous studies have concluded that high TMB is clinically beneficial because of its association with better therapeutic responses that includes higher survival rates of patients with breast cancer (Thomas et al., 2018[42]), ovarian cancer (Birkbak et al., 2013[2]), and non-small cell lung cancer (Hellmann et al., 2018[15]). However, there is little evidence as to whether or not high TMB has the same effect and is beneficial in patients with liver cancer. 

Predictive biomarkers or prognostic immune signatures of liver cancer have been well researched (Yang et al., 2011[46]; Giordano and Columbano, 2013[11]). These molecular markers are comprised primarily of non-coding RNAs such as microRNAs (miRNAs) and long noncoding RNAs (IncRNAs) (Yang et al., 2011[46]; Karakatsanis et al., 2013[18]; Zucman-Rossi et al., 2015[47]). Conceptually, some of them (e.g. miR-122, miR-145, TP53, and CTNNB1) could become ideal predictive, diagnostic or prognostic markers for specific therapeutic approaches (Karakatsanis et al., 2013[18]; Zucman-Rossi et al., 2015[47]). These signatures or biomarkers are generally associated with intratumoral immune-related cells such as T cells, NK cells, B cells, and plasma cells (Thomas et al., 2018[42]). As a result, the variation in the percentage of immune-related cells reflects the immune functions of the human body. Based on this and with innovative bioinformatics, we performed a comprehensive assessment of 1) how TMB level affects the immune-related cells, genes and miRNAs; and, 2) how miRNA/gene interaction impacts the survival (defined as 120 months to last clinical follow up or death) of LIHC patients.

## Materials and Methods

### TCGA data acquisition and sample selection

The Cancer Genome Atlas (TCGA) data (including gene mRNA expression, miRNA, mutation and clinical data sets) of patients with LIHC were downloaded from the Broad GDAC FIREHOSE website (http://gdac.broadinstitute.org/runs/stddata__2016_01_28/; TCGA data version 2016_01_28). A value of 10 somatic mutations per Mb of coding DNA corresponds to ~150 nonsynonymous mutations within expressed genes (Schumacher and Schreiber, 2015[36]). For this analysis, the TMB value per each sample was defined as the number of nonsynonymous mutations divided by 15. LIHC patients were divided into two groups, either the low TMB (< median) or the high TMB (≥ median) group.

### Immune cells percentage prediction

To describe the immune microenvironment (the immune cell characteristic matrix) for each patient, we identified the percentage of 22 immune cells per sample by R package 'EpiDISH'.

### Comprehensive genomic expression profiling

To improve the reliability of the data, we excluded genes that expressed the value of 0 in 90 % of the samples. Next, we filtered the differential expressive genes and miRNAs based on a Wilcox rank sum test and FOLD CHANGE analysis. Genes with a p < 0.01 and an FC value > 2 or < 1/2 were retained. Likewise, we only kept miRNAs with a p < 0.05 and an FC value > 2 or < 1/2. 

### Target miRNA-gene interaction

To identify the sensitivity of interaction between miRNA and genes in LIHC patient with low and high TMB, the high confidence targeting datasets were downloaded from the starBase online database (http://starbase.sysu.edu.cn/starbase2/index.php). TCGA samples were screened by the following conditions: (1) If the miRNA-gene interactive pair was derived from the starBase, the Pearson coefficient < 0 would be exclusively required; (2) If the pair was not derived, the Pearson coefficient < 0 and P-value of cor.test < 0.05 would both be required. 

### Gene functional annotation and enrichment

The gene functional annotation and enrichment analysis in terms of gene sets were performed by DAVID functional annotation tool (https://david-d.ncifcrf.gov/summary.jsp) and Fisher's Exact Test (Rivals et al., 2006[29]; Huang et al., 2008[16]; Robinson et al., 2010[30]). The equation of Fisher's Exact Test was as follows: 


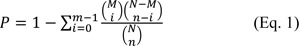


where, N is the number of all genes; n is the number of genes regulated by miRNA, M is the number of genes in a pathway, and m is the number of genes overlapped by the miRNA and the pathway. 

### Survival analyses

To further evaluate the relationship between miRNA/gene interactive pairs and the prognosis of the patients, R package {OIsurv} was used to project a patient's overall survival. Prior to all regression analysis, the range normalization between the expression values of miRNA and genes were performed according to the equation:


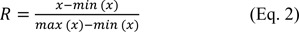


Next, Cox proportional hazards regression was fitted to obtain the risk scores (RS) for each miRNA/gene pair in low and high TMB, yielding the following equation:


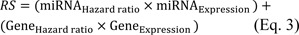


The RS allowed LIHC patients to be classified into either a low risk TMB (25 ≤ RS ≤ 75) or a high-risk TMB (RS > 75 or RS < 25) group. P-values were calculated by a Log-rank test for low TMB and high TMB patients. A combination with Cox proportional hazards regression and P-values of the Log-rank test were finally screened the significant miRNA/gene interactive pairs.

## Results

### High TMB strengthens immune-mediated infiltration

**Figure 1:**
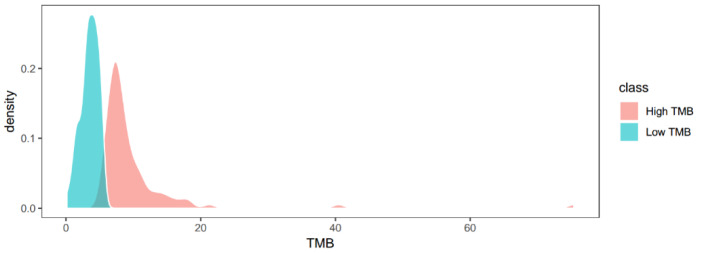
The density function of TMB value in all patients with LIHC. Low and high TMB defined by the median of TMB value, with < median as low TMB and ≥ median as high TMB

We first obtained the data set of miRNA (424 patient samples and 1047 RNAs), gene (423 patient samples and 20532 RNAs), and mutation (198 patient samples and 10230 RNAs) from TCGA. Prior to the comprehensive assessment, all the data sets were normalized for miRNAs (191 patient samples and 989 RNAs), genes (191 patient samples and 19563 RNAs), and mutations (191 patient samples and 9970 RNAs). Patients with LIHC were divided into either the low TMB (< median) or the high TMB (≥ median) group (Figure 1(Fig. 1)).

**Figure 2:**
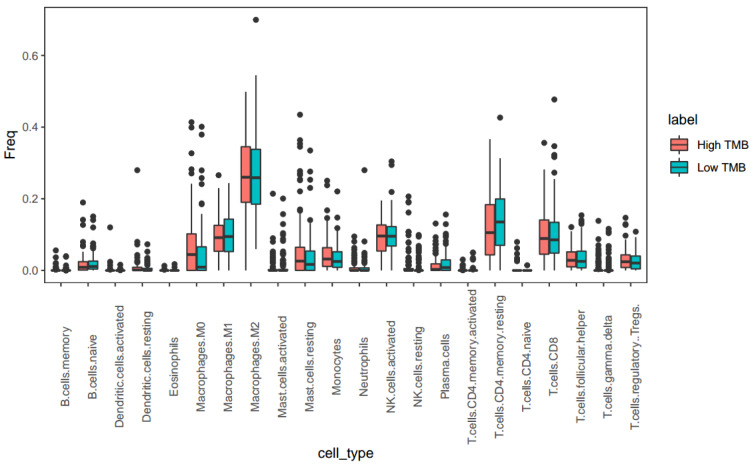
The percentage simulation of 22 immune cells between LIHC patients with low and high TMB. Based on a Wilcox rank sum test, the projected percentages of 22 immune cells were performed by the cibersort method of 'EpiDISH' package in the R environment.

We determined whether LIHC patients with high TMB could be more effectively treated with immunotherapy. The cibersort (CBS) method of 'EpiDISH', characterized by immune cells, was used to project the percentages of 22 immune cells in each patient based on a Wilcox rank sum test (Figure 2(Fig. 2)). Compared with low TMB, the percentage of plasma cell in patients with high TMB had significantly declined (Wilcox P = 0.049, FC = 0.679), but naive CD4 T-cell (Wilcox P = 0.032, FC = 20.924) were significantly elevated. There was no obvious difference in CD8 T-cell (Wilcox P = 0.569, FC = 0.903) between LIHC patients with low or high TMB. However, patients with high TMB had more memory B cell than patients with low TMB (Wilcox P = 0.053, FC = 1.760).

### The effect of TMB on immune-related genes

**Figure 3:**
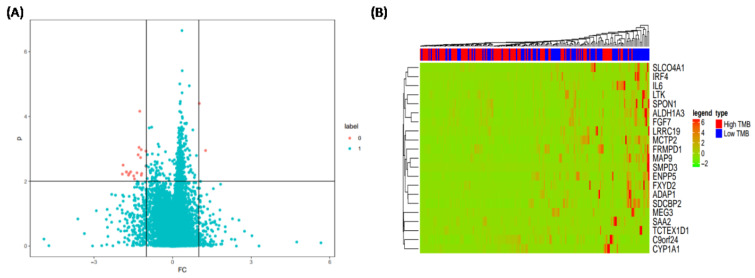
The effect of TMB on the immune-related genes of patients with low and high TMB. (A) Potential targeting genes; (B) Expressions of 21 immune-related genes

To describe how TMB responds to immune-related genes of patients with LIHC, a Wilcox rank sum test and FOLD CHANGE analysis were performed to filter the differential expressive genes. The results (Figure 3A(Fig. 3)) indicated that a total of 21 immune-related genes were obviously differentiated (*ADAP1, ALDH1A3, C9orf24, CYP1A1, ENPP5, FGF7, FRMPD1, FXYD2, IL6, IRF4, LRRC19, LTK, MAP9, MCTP2, MEG3, SAA2, SDCBP2, SLCO4A1, SMPD3, SPON1, *and *TCTEX1D1*). The genes were found in LIHC patients with low and high TMB. It is important to note that all other genes were highly expressed in patients with low TMB with the exception of *C9orf24* and *CYP1A1*. (Figure 3B(Fig. 3)). *CYP1A1* is also significantly associated with the chemical carcinogenesis pathway (p < 0.05). These results demonstrated that there are obviously different genes associated with immunity between LIHC patients with low TMB versus high TMB.

### The effect of TMB on miRNAs

**Figure 4:**
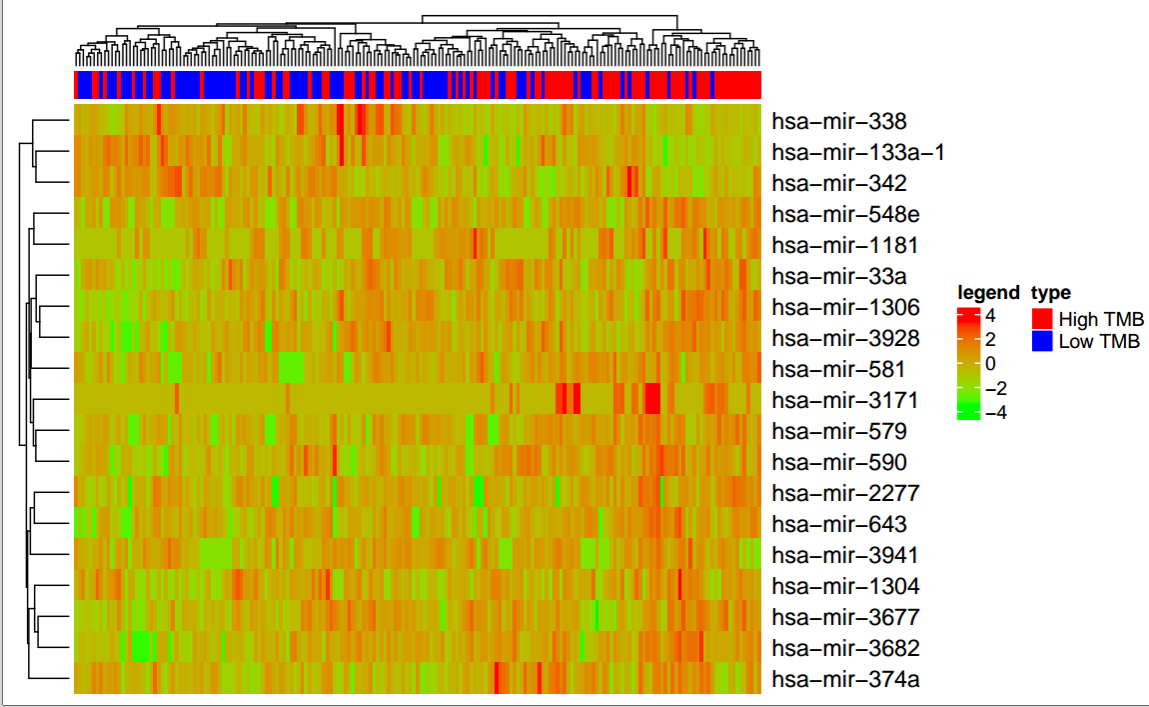
The effect of TMB on the miRNAs. Comparison of obvious differential expressions of miRNAs in LIHC patients with low and high TMB

**Figure 5:**
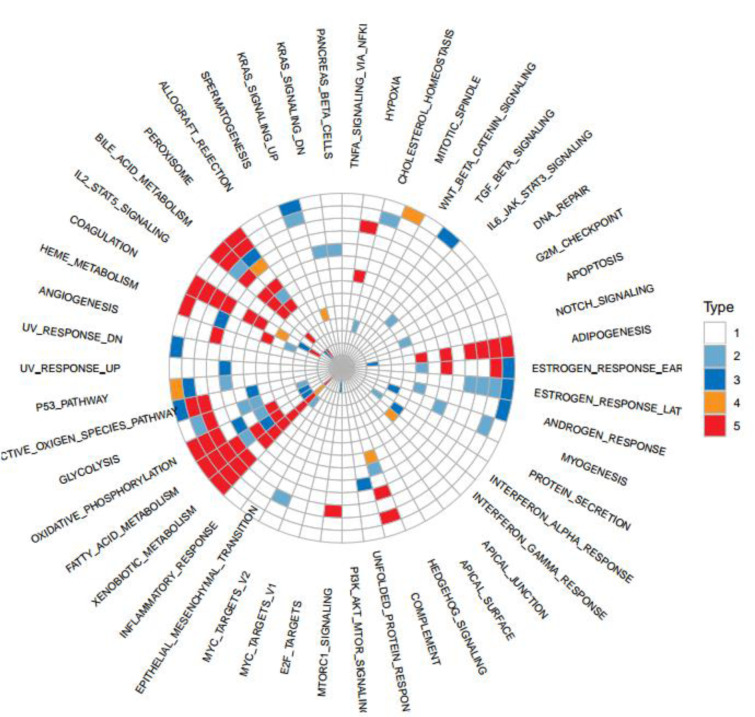
The effect of miRNAs on the functional regulation of biological processes. Functional projection associated with miRNAs was generated by the method hallmark pathway in MsigDB.

To further illuminate how miRNAs participate in the biological process of LIHC patients with low and high TMB, we screened 19 obvious differential miRNAs, including hsa-miR-338, hsa-miR-133a-1, hsa-miR-342, hsa-miR-548e, hsa-miR-1181, hsa-miR-33a, hsa-miR-1306, hsa-miR-3928, hsa-miR-581, hsa-miR-3171, hsa-miR-579, hsa-miR-590, hsa-miR-2277, hsa-miR-643, hsa-miR-3941, hsa-miR-1304, hsa-miR-3677, hsa-miR-3682, hsa-miR-374a (Figure 4(Fig. 4)). Next, we generated the miRNA/gene pairs by combining starBase, then we described the roles of these miRNAs in cancers by the method hallmark pathway in MsigDB (http://software.broadinstitute.org/gsea/msigdb) (Figure 5(Fig. 5)). These function pathways, including bile acid metabolism, estrogen response early, peroxisome, coagulation, oxidative phosphorylation, xenobiotic metabolism, adipogenesis, and fatty acid metabolism, were significantly associated with miRNAs if Fisher's Exact Test was confirmed.

### Relationship between the miRNA/gene pair and patient survival

**Figure 6:**
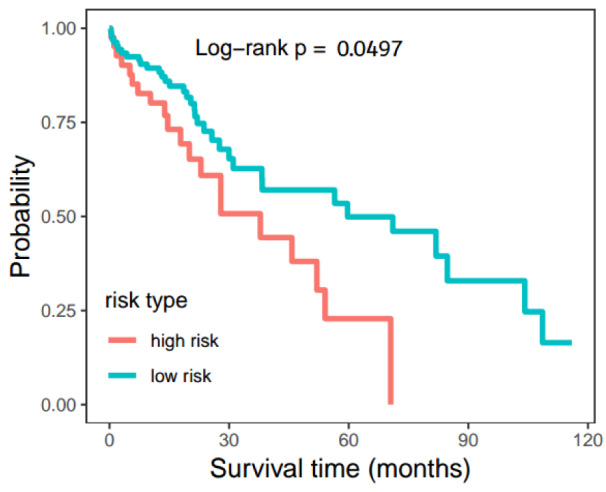
The overall survival rate projection of LIHC patients. The Hsa-miR-33a/*ALDH1A3* pair was used to project the survival time of LIHC patients by Cox proportional hazards regression and Log-rank test, the p-value obtained from the Log-rank test.

To investigate the clinical relevance of survival and TMB, Cox proportional hazards regression and Log-rank test were used to fit associations between molecular variables (miRNA/gene pair) and survival time. For each miRNA/gene pair, we only kept the pairs that contained disparities in both the miRNA and gene for the survival analysis. As a result, 8 significant miRNA-gene pairs were found, including hsa-miR-33a/*SPON1*, hsa-miR-33a/*ADAP1*, hsa-miR-33a/*ALDH1A3*, hsa-miR-1306/*SAA2*, hsa-miR-3928/*SAA2*, hsa-miR-3677/*SAA2*, hsa-miR-3682/*SAA2* and hsa-miR-374a/*MEG3*. Using the Log-rank test, the hsa-miR-33a/*ALDH1A3* was significantly associated with a poor prognosis of LIHC patients (p < 0.05). The formula of survival model was Risk Score = (-0.34459987) * hsa-miR-33a + (-0.05001263) * *ALDH1A3* [Log-rank p = 0.0497, (HR = 1.702, 95 %CI:0.9948-2.912)]. Based on this, we predicted the effect of the hsa-miR-33a/*ALDH1A3* pair on the overall survival of LIHC patients (Figure 6(Fig. 6)) and that the survival of LIHC patients would be more than 60 months from the initial clinical dates in either the high risk or the low risk group.

## Discussion

Immunotherapy as an effective therapeutic intervention has become extensively accepted to treat patients with cancer, and is more effective than current vaccinations (Rosenberg et al., 2004[31]). More notably, TMB is becoming an effective intervention, thus leading immuno-oncology as precision medicine therapies (Steuer and Ramalingam, 2018[39]). Concern regarding the predictive effect of TMB on the cancer microenvironment has led to substantial research of predictors or prognostic biomarkers (Chalmers et al., 2017[5]; Goodman et al., 2017[12]). Some studies indicate that cancer patients with high TMB can easily benefit from immunotherapy (Birkbak et al., 2013[2]; Gubin et al., 2015[13]; Thomas et al., 2018[42]). In an intratumoral immune microenvironment, the presence of different immune cell lineages, such as T cells/NK cells (T/NK metagene), B cells/plasma cells (B/P metagene), and Myeloid/Dendritic cells (M/D metagene) have direct positive roles on immune infiltrates, thus increasing the survival rates of cancer patients (Thomas et al., 2018[42]). 

In this study, we investigated the percentage variation of the immune-related cells in LIHC patients with low and high TMB. Our results indicate that patients with high TMB had more CD4 T-cells, rather than plasma cells and CD8 T-cells. The number of memory B cells in LIHC patients with high TMB was significantly higher when compared with patients with low TMB. As a result, we concluded that the innate immune system is more active than an acquired immunity in LIHC patients with high TMB. Immunologic tolerance is supported by regulatory CD25+ and CD4+ T cells controlling autoimmunity, tumor immunity, and transplantation tolerance (Sakaguchi et al., 2001[34]). Activation of CD4 T-cells in a cancer microenvironment also impacts the immunity system. In other words, a protective benefit of high TMB is that it improves the immune system of LIHC patients. This is a potential explanation for why cancer patients with high TMB are more likely to benefit from immunotherapy and why cancer patients with low TMB show limited benefits from Neoantigen-targeted vaccines (Martin et al., 2016[23]). Therefore, our study points to the fact that tumor cells may be directly phagocytozed by NK cells without relying on plasma cells and CD8 cell intervention. Moreover, 21 immune-related genes were obviously different between LIHC patients with low and high TMB. Elevated *C9orf24* and *CYP1A1* were found in LIHC patients with high TMB. *CYP1A1* gene encodes a member of the cytochrome P450 superfamily of enzymes (Nebert et al., 1987[26]). The cytochrome P450 proteins are monooxygenases that catalyze many reactions involved in drug metabolism and synthesis of cholesterol, steroids and other lipids (Hannemann et al., 2007[14]). This protein localizes to the endoplasmic reticulum and its expression is induced by some polycyclic aromatic hydrocarbons (PAHs) (Shimada et al., 2002[38]); some PAHs are found in cigarette smoke. The enzyme's endogenous substrate is unknown; however, it is able to metabolize some PAHs to carcinogenic intermediates. The *C9orf24 *gene has been associated with lung cancer risk and encodes ciliated bronchial epithelium 1 (Ross et al., 2007[32]). However, there is little evidence as to the mechanism of *C9orf24* gene. 

In addition, these differential immune genes are associated with various key biological processes, such as GTPase activator activity, T-helper 17 cell lineage commitment, defense response to protozoan, and response to antibiotics. For example, *ADAP1* (ArfGAP With Dual PH Domains 1) is a GTPase-activating protein (GAP), related to the pathways including B cell receptor signaling pathway (sino) and *Arf6 *signaling events via GTPase activator activity and inositol 1,3,4,5 tetrakisphosphate binding (Stricker et al., 2006[40]; Giguere et al., 2018[10]). *IL6* gene encodes a cytokine that functions in inflammation. The maturation of B cells and its encoded protein has an endogenous pyrogen capable of inducing fevers in people with autoimmune diseases or infections (Tanaka et al., 2014[41]). This protein is primarily produced at acute and chronic inflammation sites, where it is secreted into the serum and induces a transcriptional inflammatory response through interleukin 6 receptor (IL-6), alpha. IL-6 is implicated in a wide variety of inflammation-associated diseases including diabetes mellitus and systemic juvenile rheumatoid arthritis (Fishman et al., 1998[9]). *TGF-β* and *IL-6* drive the production of *IL-17*, further regulating T-helper 17 (Th17) (McGeachy et al., 2007[24]).

In relation to miRNAs, we observed 19 obviously differential miRNAs between LIHC patients with low and high TMB. We found that these miRNAs are associated with some function pathways of metabolic energy balance and immune responses. Some miRNAs (hsa-miR-133a-1, hsa-miR-1181, and hsa-miR-3171) are related to the formative process of liver cancer (Chen, 2009[7]; Murakami and Tanahashi, 2013[25]; Lu et al., 2014[21]). Other miRNAs are involved in the cell proliferation and invasion. For example, miRNA-338 suppresses gastric cancer cell growth, invasion, and metastasis via targeting *NRP1* expression (Peng et al., 2014[27]). miR-374a regulates Pre-B-cell colony-enhancing factors in human lung endothelium (Adyshev et al., 2014[1]). Moreover, these miRNA-mediated functions (e.g. fatty acid metabolism, adipogenesis, and oxidative phosphorylation) are associated with liver function (Polimeno et al., 2000[28]; Ross et al., 2002[33]; Kohjima et al., 2007[19]). As a result, we documented the importance of miRNAs in developing a treatment for LIHC.

In this study, we found that the pair hsa-miR-33a/ALDH1A3 significantly prolongs the survival of LIHC patients. The miRNA/gene relationship was frequently utilized to predict the stage, size, and prognosis, offering a new therapeutic strategy for cancers. In this context, the pair hsa-miR-33a/*ALDH1A3* axis correlated positively with TMB. High expression of hsa-miR-33a/*ALDH1A3* had a corresponding better prognosis and the up-regulation significantly prolonged the survival rate of LIHC patients. As an independent prognostic marker, *ALDH1A3* has been reported to have a positive impact in the overall survival time of cancer patients (e.g., NSCLC patients) (Shao et al., 2014[37]), whereas elevated *ALDH1A3* was associated with worse prognosis in triple-negative breast cancers (Marcato et al., 2015[22]). Has-miR-33a was recently identified as a regulator in the development of liver cancer. Liu et al. (2018[20]) determined that miR-33a-5p negatively regulated the expression *of PNMA1* to control the cell proliferation in hepatocellular carcinoma (HCC) via the Wnt/β-catenin pathway. Unfortunately, the regulative mechanism of has-miR-33a is still unclear. A recent review suggested that *ALDH1A3* targeting miRNAs is strongly associated with the development and prognosis of malignant tumors (Duan et al., 2016[8]). For example, the expression of *ALDH1A3 *was regulated by the binding between miR‐125a/b and 3'UTR of *ALDH1A3* that could attenuate cell proliferation and enhance cell apoptosis (Chen et al., 2013[6]). The joint effect was also confirmed in *miR-187* and *ALDH1A3. MiR-187* is also directly associated with the development of prostate cancer (Casanova-Salas et al., 2015[4]). Our findings predicted the roles of *ALDH1A3* in LIHC, although there is no empirical evidence to explain this. 

In conclusion, low and high TMB are associated with the response of immune-mediated cells, miRNAs and gene expression. High TMB strengthens immune-mediated infiltration. The miRNA/gene pair, hsa-miR-33a/*ALDH1A3*, may become a potential prognostic marker in LIHC patients. Our results suggest that high TMB are powerful prognostic biomarkers; therefore, LIHC patients with high TMB could benefit from immunotherapy.

## Notes

Xiao-Ping Mei and Tai-Yong Fang (Department of Gastroenterology, Second Affiliated Hospital, Fujian Medical University, 34, Zhongshan North Road, Quanzhou 362000, China; E-mail: fangtaiyong@126.com) contributed equally as corresponding authors.

## Conflict of interest

The authors have no conflict of interest to report.

## Acknowledgements

Not applicable.
